# A history of childhood schizophrenia and lessons for autism

**DOI:** 10.1007/s40656-024-00627-5

**Published:** 2024-08-12

**Authors:** Sam Fellowes

**Affiliations:** Department of Politics, Philosophy and Religion, County South, Lancaster University, Lancaster, LA1 4YL UK

**Keywords:** Childhood schizophrenia, Autism spectrum disorders, Child psychiatry, Diagnostic criteria, Causally formulated diagnoses

## Abstract

The diagnosis of childhood schizophrenia was widely employed in the U.S. from the 1930s to the late 1970s. In this paper I will provide a history of the diagnosis. Some of the earliest publications on childhood schizophrenia outlined the notion that childhood schizophrenia had different types. I will outline the development of these types, outlining differing symptoms and causes associated with various types. I outline how different types of childhood schizophrenia were demarcated from one another primarily on age of onset and the type of psychosis which was believed to be present. I will outline how various child psychiatrists viewed the types of childhood schizophrenia posited by other child psychiatrists. I will outline the process of abandoning childhood schizophrenia. I use my history to challenge what I believe are misconceptions about childhood schizophrenia. Also, I will use my history to draw lessons for thinking about modern notions of autism. It shows potential problems around formulating psychiatric diagnoses around causes and how compromises might be needed to prevent those problems. Additionally, childhood schizophrenia shows that psychiatrists could formulate subtypes that are not based upon functioning levels and that we can conceive of subtypes as dynamic whereby someone can change which subtype they exhibit over time.

## Introduction

The diagnosis of childhood schizophrenia was widely employed in the U.S. from the 1930s to the late 1970s. Histories of autism sometimes also contain material on the diagnosis of childhood schizophrenia. This is because there are significant links between both diagnoses. Childhood schizophrenia is partially an historical precedent for autism. DSM-5 Autistic Spectrum Disorder (henceforth autism) has been built out of and upon earlier notions of DSM autism. In the DSM-III, the diagnosis of autism was explicitly introduced to replace childhood schizophrenia (APA, 1980, p. 86 and p. 375). Also, as I will show, the symptoms associated with childhood schizophrenia fit DSM 5 autism closer than any other DSM 5 diagnosis. However, there has yet to be a dedicated paper providing a history of childhood schizophrenia. In this paper I will provide this history and use it to challenge what I believe are misconceptions about childhood schizophrenia. Also, I will use my history to draw lessons for thinking about modern notions of autism.

My main aim is to outlined the development of different types of childhood schizophrenia. Some of the earliest publications on childhood schizophrenia outlined the notion that childhood schizophrenia had different types. I will outline the development of these types, outlining differing symptoms and causes associated with various types. I outline how different types of childhood schizophrenia were demarcated from one another primarily on age of onset and the type of psychosis which was believed to be present. Also, I will outline how various child psychiatrists viewed the types of childhood schizophrenia posited by other child psychiatrists. Finally, I will outline the process of abandoning childhood schizophrenia. For reasons of brevity and to make focused points, I only discuss north American notions of childhood schizophrenia. There may well have been differences between notions of childhood schizophrenia employed in north America and in the UK and Continental Europe. As yet, there has been little historical investigation comparing north American notions of childhood schizophrenia with British or continental notions, but child psychiatrists writing in the period sometimes mentioned differences (for example, Kanner, 1965, p. 413; Kolvin, 1972, p. 816; Rutter, 1972, p. 329).

My history shows that there was a significant level in nuance in how childhood schizophrenia was conceptualised. This challenges the claim that “[c]hildhood schizophrenia… [was] stretched so thin as to become meaningless” (Eyal et al., 2010, p. 131) and critiques the claim that childhood schizophrenia and associated diagnoses were used “interchangeably” (Silverman, 2012, p. 39; Raz, 2014, p. 3).

I will draw lessons from my history for thinking about modern notions of autism. Given that childhood schizophrenia and DSM 5 autism cover significantly overlapping symptoms we can use childhood schizophrenia to think about alternative ways of formulating diagnoses to cover the symptoms associated with DSM 5 autism. Childhood schizophrenia was causally demarcated. The supposed presence of the cause meant that the diagnosis was considered present. However, this then meant childhood schizophrenia covered a broad clinical picture. I show that the clinical picture of autism might also be expanded if causally defined. I show how value decisions and potential compromises might be needed to prevent this. Also, childhood schizophrenia shows how diagnoses can be formulated around dynamic causes whereby the causes present in an individual might change over time. This is a relevant point given recent understanding of causes associated with autism. Additionally, childhood schizophrenia shows that psychiatrists could formulate subtypes that are not based upon functioning levels and that we can conceive of subtypes as dynamic whereby someone can change which subtype they exhibit over time.

I start with a literature review. Then I outline the key steps in the development of concepts of childhood schizophrenia in the U.S. Then I outline the abandonment of childhood schizophrenia. Finally, I consider how childhood schizophrenia provides lessons for thinking about modern notions of autism.

## Literature review

Discussion of historical notions of childhood schizophrenia usually take place within histories of autism. Whether childhood schizophrenia is mentioned typically depends upon how historical scholarship approaches ‘autism’. This usually takes one of three forms. Firstly, ‘autism’ can be whichever historical diagnosis, regardless of its name, looks closest to recent DSM notions of autism. Secondly, ‘autism’ can be the theoretical concept named autism which was employed between the 1910s to the 1960s. Thirdly, ‘autism’ can be the psychiatric diagnosis named autism which was employed between the 1940s to the 1970s. My history of childhood schizophrenia contributes to all three approaches.

Following the first approach, the symptoms attributed to childhood schizophrenia had significant similarities to those attributed to DSM-5 autism. Symptoms included “specific thinking and perceptual disorder, mental deterioration, specific language and speech disorder, dissociation of affect” (Despert, 1941, p. 532). This bares parallel with autism where children are thought to have cognitive, perceptual, social, language and emotional abnormalities, whilst some also have low intellect. Also, many symptoms are specifically associated with autism. For example, childhood schizophrenic individuals dislike being interrupted, they follow rituals, they have compulsions, they are sensitive to criticism (Bradley, 1941), they dislike novel situations, some have co-ordination problems, some are skilled in the arts, some are good at abstract thinking, they sometimes cannot transfer abilities from one environment to another (Bender, 1947), they exhibit non-functional play, they do not use the third person and they sometimes have a monotone voice (Despert, 1965 [1947]). Additionally, unlike modern notions of schizophrenia, childhood schizophrenics were typically considered to not hallucinate (Alderton, 1966, p. 282; Bender, 1947, p. 50; Ornitz & Ritvo, 1968, p. 78; Robinson, 1961, p. 544). Without suggesting it is identical this list of symptoms looks closer to autism than any other DSM-5 diagnosis. I provide more details upon the symptoms associated with childhood schizophrenia than is present in any existing historical account.

Following the second approach, Evans has argued the meaning of the term autism has undergone a “radical transformation” (2017, p. 11). During the 1910s until the 1960s autism was conceptualised as a retreat from interacting with the external world and an immersion in a life of fantasies. During the 1960s autism was reformulated into a statistical notion which described (supposedly) observable behaviours. It is from this reformulated notion that DSM-5 autism can be traced. Evans provides an insightful history about causal theories which were applied to both historical notions of autism and childhood schizophrenia. I can contribute to this by outlining different types of childhood schizophrenia and show some of those types were associated with more specific fine-grained causes. Also, Evans primarily, though not exclusively, focuses upon British sources whereas I focus exclusively upon north American sources.

Following the third approach, there was a diagnosis called autism formulated by Leo Kanner which has received considerable historical scholarship (for example, Eyal et al., 2010; Jacobsen, 2010; Verhoeff, 2013b). Historical scholarship has outlined how Kanner sometimes saw autism as distinct from childhood schizophrenia and sometimes closely associated them. I can contribute to this by outlining how Kanner and other child psychiatrists positioned the diagnosis of autism within the other types of childhood schizophrenia being employed.

My argument will challenge some recent claims about childhood schizophrenia. Eyal et al. accurately identify childhood schizophrenia as covering many similar symptoms to DSM-IV autism. However, Eyal et al. argue childhood schizophrenia expanded too far by covering too many diverse clinical pictures. This left “[c]hildhood schizophrenia… stretched so thin as to become meaningless” (Eyal et al., 2010, p. 131). They do not define ‘meaningless’ but given their concern relates to the diversity of clinical pictures it seems to mean ‘provide useful clinical pictures’. Whilst childhood schizophrenia did cover a wider range of symptoms than that associated with DSM 5 autism, and the diagnosis arguably did cover too diverse clinical pictures, I will show how childhood schizophrenia and its types conveyed more restrictive and more specific clinical information than Eyal et al. believe.

It has also been claimed autism and childhood schizophrenia were used interchangeably. Silverman writes that “most followed Kanner’s lead and used the terms “childhood schizophrenia” and “autism” interchangeably” (2012, p. 39) and Raz writes “[u]ntil the early 1970s, diagnoses as divergent as the ‘atypical child’, childhood psychosis, schizophrenia and infantile autism were used interchangeably by some researchers” (Raz, 2014, p. 3). This is true of some child psychiatrists, but I will outline how major figures within child psychiatry explicitly demarcated different types and did not use them interchangeably.

Finally, my argument is also relevant to historical scholarship discussing the possibility of alternative formulations of autism. Verhoeff suggests DSM-IV autism should be replaced (2013a, p. 9) and Fellowes suggests historical notions of autism and childhood schizophrenia warrant investigation for a source of alternative diagnoses (2017, p. 58). Multiple critics have been concerned that the diagnosis of autism is too heterogeneous both in associated symptoms and underlying causes (Cushing, 2013, p. 38; Hassall, 2016, p. 51 Timini et al., 2011, p. 7; Verhoeff, 2013a, p. 9), a concern that is also associated with most DSM diagnoses (Cuthbert & Insel, 2013). I will later suggest that we can use my history of childhood schizophrenia to think about alternative approaches to DSM 5 autism.

## The development of different types of childhood schizophrenia

Prior to the 1920s in U.S. psychiatry there was no separate child psychiatry (Kanner, 1959, p. 582) with children being diagnosed instead according to adult diagnoses (Bradley, 1941, p. 19). Kanner’s 1935 text book *Child Psychiatry* (the first English language textbook on child psychiatry) includes nineteen pages on schizophrenia within a chapter on major psychosis. This section only discusses adult symptoms (Kanner, 1935, pp. 484–492) and has no symptoms specific to children. The only difference mentioned is that the children typically have a more variable profile compared to adults but what these profiles were is not mentioned. However, whilst Kanner was writing his textbook a new notion of childhood schizophrenia with symptoms different to adult symptoms was being developed. Whilst absent from Kanner’s, 1935 textbook, Kanner later identified Potter (1933) as the first to formulate a new notion of childhood schizophrenia that soon became a major notion in child psychiatry (Kanner, 1971, p. 17).

### Early developments

Howard W Potter was the Assistant Director of New York Psychiatric Institute and Hospital. His 1933 article *Schizophrenia in Children* appeared in the *American Journal of Psychiatry*. He takes childhood schizophrenia as covering “only such persons who show no physical evidence of puberty and are largely in the first decade of life” (Potter, 1933, p. 1254). Potter outlined, in list format, the symptoms he thought “characterized” childhood schizophrenia:

1. A generalised retraction of interest from the environment.
2. Dereistic [fantasy] thinking, feeling and acting.
3. Disturbance of thought, manifested through blocking, symbolization, condensation, perseveration, incoherence and diminution, sometimes to the extent of mutism.
4. Defects in emotional rapport.
5. Diminution, rigidity and distortion of affect.
6. Alternations of behaviour with either an increase of motility, leading to incessant activity, or a diminution of motility, leading to complete immobility or bizarre behavior with a tendency to perseveration or stereotypy (Potter, 1933, p. 1254).

Based upon the case studies he provides it appears that childhood schizophrenia requires only some, rather than all, these symptoms. Potter considers these children to be psychotic, having an abnormal relationship with reality.

[W]hat little delusional formation there is, is exceedingly simple and naïve. The outstanding symptomatology is found in the field of behaviour and a consistent lack of emotional rapport. The drive for integration with the environment, so characteristic of normal children and so essential for their personality development is outstandingly absent (Potter, 1933, p. 1268).

There is, however, no clear definition of psychosis.

Through the historical period I cover psychosis was typically conceptualised as an abnormal relationship with the external world which made the child struggle to interact with the external world (see Evans, 2017 for details). Consequently, the child would withdraw from the world. A common interpretation conceptualised psychosis in psychoanalytical terms whereby various defence mechanisms which mediated the relationship between the child and the external world failed to develop normally. Opinion varied as to what caused psychosis. Some child psychiatrists thought poor mothering resulted in abnormal ego development but most thought a combination of biological and environmental factors were required and that poor mothering was only one possible environmental factor. Each patient with childhood schizophrenia was considered to have psychosis and thus the diagnosis was causally demarcated.

Psychosis generally appeared to be understood as both a characteristic which was caused by various defence mechanisms and as a cause which produced further symptoms. Psychosis was the characteristic of withdrawing but this withdrawal would then cause “secondary” (Bender, 1953, p. 676) or “accessory” (Mahler, 1965, p. 557) symptoms. This situation is largely analogous to modern cognitive psychological theories of autism. For example, theory of mind deficits are taken as being a characteristic which describes difficulty with seeing other perspectives. This characteristic is taken as caused by underlying biology but, at the same time, researchers believe that theory of mind deficits then cause various symptoms such as difficulty understanding the social world (Rajendran & Mitchell, 2007, p. 231).

Potter only briefly mentioned how childhood schizophrenia relates to other diagnoses. In his case-studies Potter often mentions his thoughts about the presence or absence of organic abnormalities but does not explicitly state whether childhood schizophrenia can be caused by or co-morbid with organic abnormalities. Also, he says there is a “superficial resemblance of schizophrenic children to certain so-called unstable mental defectives” (Potter, 1933, p. 1268) but does not state whether schizophrenia and mental deficiency can be co-morbid. Other child psychiatrists would soon expand on Potter’s account.

After Potter’s initial description the second important step in the development of U.S. concepts of childhood schizophrenia was the development of the notion that there might be different types of childhood schizophrenia. These types which were demarcated by type of onset. Initially, these ideas were developed by Louise J Despert and Charles Bradley (see Kanner for similar and slightly earlier demarcation made by European psychiatrists (Kanner, 1965, p. 417).

Louise J Despert was a New York based child psychiatrist and a major figure working on childhood schizophrenia. Kanner, in the 2nd edition of his textbook *Child Psychiatry*, references more articles by Despert than anyone else (1948, p. 729). She initially outlined her approach to childhood schizophrenia in a 1938 article entitled *Schizophrenia in Children* published in *Psychiatric Quarterly*. The paper describes children admitted to the New York State Psychiatric Institute.

Despert demarcated two types of onset of childhood schizophrenia. There is insidious onset, where the illness slowly develops over time, and acute onset, where the illness develops very suddenly (she also describes a third group, that of insidious onset followed by acute episodes, but this is generally not discussed by later psychiatrists).^1^ Despert was the first American psychiatrist to demarcate between different types of childhood schizophrenia on the basis of type of onset, though some European psychiatrists had previously made similar demarcations (see for discussion Kanner, 1965, p. 417).

Despert does not explicitly specify that each type of childhood schizophrenia has distinct symptoms. Instead she lists symptoms which she noted as present within her patients. Those with schizophrenia of acute onset had a worse prognosis (1938, p. 368).^2^ Only children with schizophrenia of acute onset (and those with insidious onset followed by acute episodes) were mute whilst none of those with a disorder that began insidiously were mute. Children with acute-onset schizophrenia often had low intellect whereas this was not true of those with insidious onset schizophrenia (1938, p. 369). Children with acute onset schizophrenia regressed, “expressed by a reversal to very primitive forms of behaviour; they soil, manipulate faeces and sputum, and some go as far as to eat garbage” (1938, p. 369).^3^ There are far fewer details about those with insidious onset schizophrenia. The most concrete characterisation is that acute onset schizophrenia ultimately resulted in a “lowering of the ideo-affective level” (1938, p. 367). Despert’s, 1938 paper only briefly touches upon demarcation criteria from other disorders or the possibility of co-morbidity but she believes some children diagnosed as feebleminded might actually be schizophrenic (Despert, 1938, p. 371). A few years later she listed differential criteria. “Severe behavior disorders, associated with regressive characteristics, are not uncommon in young children with acute emotional disturbances. However, in the absence of affective dissociation [psychosis], the diagnosis of schizophrenia cannot be made, however severe the behavior disorder” (Despert, 1941, p. 536). In her view, two individuals might exhibit similar symptoms but only those with underlying psychosis are schizophrenic.

The demarcation Despert makes between schizophrenias with acute and insidious onset was made more explicit in Charles Bradley’s book length literature review titled *Childhood Schizophrenia* (1941). The book primarily reviews European literature; Potter and Despert are the main American sources. Bradley makes each grouping more concrete by listing typical symptom profiles of both types of onset rather than, like Despert, simply listing symptoms observed in patients with each onset. Acute childhood schizophrenia occurs “in a sudden, stormy fashion” (1941, p. 38). The acute course is characterised by psychomotor manifestations, active negativism, extreme irritability, pressure of speech, dramatic catatonic manifestations such as posturing, grimacing, peculiar gait (1941, p. 39). This phase can last days or weeks (1941, p. 39). When it fades the child never fully recovers to the pre-acute phase and ends up “lacking the normal childhood spontaneity and freedom of reaction to his environment which characterised his early childhood” (1941, p. 39). Insidious onset schizophrenia “develops slowly and insidiously and often makes itself evident by a gradual loss of interest in the surroundings. There may be signs of withdrawal from the usual group activities of children and quiet preoccupation with the patient’s own thoughts and solitary interests. Eventually as the result of this the child fails to adapt to his surroundings sufficiently to warrant the diagnosis of psychosis” (1941, p. 43). This decline has a variable rate (1941, p. 43). It might reach the stage where the child ceased reacting to the environment, or the decline might stop far before that (1941, p. 43). Those with acute-onset schizophrenias, and those with an earlier age of onset, had a worse prognosis (1941, p. 121).

Bradley demarcated childhood schizophrenia from mental deficiency, organic disorders and non-psychotic neurosis. Schizophrenic children sometimes declined to intellectual levels comparable with mental retardation (to use the historical term) (1941, pp. 54–55) whilst mental retardation often had some symptoms similar to childhood schizophrenia (1941 p. 94). Therefore, onset of illness is key to differentiating mental retardation and childhood schizophrenia (1941, p. 81). Bradley, however, also suggests mental retardation and schizophrenia can co-exist (1941, p. 55) and that both should be diagnosed if present (1941, p. 95). He demarcates childhood schizophrenia from neurosis (1941, p. 102), prepsychotic, schizoid and schizoid psychopaths who can resemble childhood schizophrenics but do not have psychosis (1941, p. 92). Bradley offers two criteria for differential diagnosis, the presence of psychosis and the illness being not present from birth.

Despert and Bradley made significant additions to Potter’s notions of childhood schizophrenia. They employed psychosis as differential criteria from other diagnoses and outlined two different types of childhood schizophrenia. They believed that similar clinical pictures could be found in other diagnoses but unless psychosis was present it was not childhood schizophrenia.

The notion that childhood schizophrenia always developed after some period of normal development was challenged by Leo Kanner, a child psychiatrist who worked in John Hopkins university. In 1943 Leo Kanner introduces the diagnosis of early infantile autism. This is “an *extreme autistic aloneness*, that, whenever possible, disregards, ignores, shuts out anything that comes to the child from the outside” (Kanner, 1943, p. 242, emphasis original). These children live in their own world, actively shunning human contact and disliking intrusions into their world. In that 1943 paper Kanner does not consider autism to be a type of childhood schizophrenia since, unlike Potter’s, Despert’s and Bradley’s notion of childhood schizophrenia, Kanner considers autism to be present from birth (1943, p. 248). Kanner, however, soon changed his views. His 1949 paper considers autism a type of childhood schizophrenia. Unlike his 1943 paper, “[t]he basic nature of its [autism’s] manifestations is so intimately related to the basic nature of childhood schizophrenia as to be indistinguishable from it’ (Kanner, 1949, p. 419) except for being present from birth. (Kanner, 1949, p. 149). He later stated that whilst autistic children share many symptoms of childhood schizophrenia, they have the specific symptoms of aloneness and desire for sameness and they usually have a less severe clinical picture (Kanner & Lesser, 1958, p. 728). Unlike in 1943, Kanner now considered autism a type of childhood schizophrenia which did not decline from normality.

Thus, by the late 1950s, in Kanner’s view, childhood schizophrenia could be present from birth (autism), it could have a sudden onset (acute childhood schizophrenia) and it could have a slow onset (insidious childhood schizophrenia). Each different type had different associated symptoms alongside many overlapping symptoms.

### Splitting by form of psychosis

Until the late 1940s, child psychiatrists distinguished childhood schizophrenia from other diagnosis by the presence of psychosis and distinguished different types of childhood schizophrenia on the basis of age and course of onset. In 1949, the New York based Hungarian child psychiatrist Margaret Mahler suggested that different types of psychosis might produce different types of childhood schizophrenia. Mahler sought to distinguish a new type of childhood schizophrenia - symbiotic psychosis. She did not list specific symptoms or diagnostic criteria but instead defined symbiotic psychosis on the basis of the supposed underlying psychoanalytical cause. She believed the children did not further develop from the mother-infancy dual unity, meaning they had a “lack of emotional separation or differentiation from the mother” (Mahler, 1952, p. 297). She saw this as a form of childhood schizophrenia which had a delayed onset. She thought symbiotic psychosis started when the normal step of the child conceptualising themselves as separate from the mother did not manifest.

Symbiotic psychosis contained a new conceptual development. Despert, Bradley and Kanner thought all individuals diagnosed with childhood schizophrenia had an underlying psychosis. The different types they exhibited depended upon the time and nature of onset. In contrast, Mahler demarcates a new type of childhood schizophrenia not on time or age of onset but rather on the specific defense mechanisms activated which result in a symbiotic form of psychosis, whereas different defense mechanisms would lead to autism, acute onset or insidious onset.

In the late 1940s New York based child psychiatrist Lauretta Bender demarcated three different types of childhood schizophrenia based upon the “pattern of the psychosis” (1953, p. 678). These were intended to incorporate the notions of childhood schizophrenia I have previously described. She believed that “[i]n the classification of childhood schizophrenia, the most important factor is the age of onset of the illness. The second factor is the progression or severity of the illness, whether it is rapid and profound or slow and slight, accelerating or regressive, and if the progression is steady or subject to remissions or arrests” (1947, p. 53). This approach which factored into account onset and progression formed the basis of her demarcation between three types of clinical pictures (1947, pp. 53–55). These are further developed in a 1956 paper whereby she outlines three different types. She believed.

Three types of clinical pictures are presented depending in part upon the defence mechanism: (1) the pseudodefective or autistic, retarded or inhibited child; (2) the pseudoneurotic with any number of neurotic mechanisms and evident activity; (3) the pseudopsychopathic with paranoid ideation, and a tendency to act out in anti-social behaviour (1956, p. 499).

Pseudodefective, the first type, “may have been retarded in maturation from the beginning or may have regressed after an early nearly normal development… He is repressed, inhibited, withdrawn, often mute and incapable of adequate object relation” (1956, p. 449). This is a severe clinical picture. Pseudodefectives might be present from birth or may have an early onset. Pseudoneurotics, the second type, may “have disturbed thought processes, disturbed speech, sensory distortions, exaggerated or unusual interjections and projections; they often have exaggerated insight, and an exaggerated capacity to relate and often with high verbal, graphic capacities and other symbol formations and high intelligence” (1956, p. 449). This can cover higher functioning children than pseudodefectives and is “most frequently seen in mid-childhood” (1959a, p. 492). Psudeopsychopathics, the third type, “tend to aggressive and anti-social acting-out behaviour. They wander about aimlessly or with compulsive and obsessional patterns such as counting street numbers, following the sky line of the city or riding all the subways… they are impulsively aggressive, fire-setters and nonconformist. They lack insight and feel no guilt or anxiety” (1956, p. 500). Communication issues are less emphasised, they have a narrower clinical picture and are less impaired than pseudoneurotics or especially pseudodefectives. Pseudopsychopathic occurs “in late childhood or early puberty” (1956, p. 500). I have previously suggested that childhood schizophrenia covered many symptoms associated with DSM 5 autism. Whilst this is also true of Bender’s approach, it is also evident that notions of childhood schizophrenia also covered a significantly broader range of symptoms than DSM 5 autism.

Like Mahler, Bender formulates her different types based upon underlying causes. Bender sees multiple causal factors being present within childhood schizophrenia, but some causes are more prominent in each type. In her 1956 paper she writes that “[t]hree types of clinical pictures are presented depending in part upon the defense mechanisms” (1956, p. 499). She later described childhood schizophrenia as “precipitated by an early physiologic or organic crisis and a failure in adequate defense mechanism… [childhood schizophrenia] exhibit[es] different clinical or behavioral or psychiatric features at different epochs in the individual’s development and in relationship to compensating defenses” (1970, p. 165). A failure in defense mechanisms need occur but which type of childhood schizophrenia is present depends on the type of defense mechanisms.

Bender makes an additional conceptual innovation. Bender sees these different types as depending on dynamic processes which can change over time. As the psychosis changes, so too do the symptoms exhibited and the applicable type also. The child can exhibit one type and then change to another type. Bender believed onset could start at any stage. A child might go through all three stages by starting pseudodefective, then later become pseudoneurotic and then become pseudopsychopathic as they age. They might start pseudodefective and remain there, or develop into pseudoneurotic. They can start and remain pseudoneurotic or develop into pseudopsychopathic. Finally, they could start pseudopsychopathic, thereby avoiding the earlier stages. Bender’s approach covered multiple ages of onset and children whose symptoms changed over time.

Bender took her approach to childhood schizophrenia as encompassing other notions of childhood schizophrenia. She took Kanner’s autism as being an instance of pseudodefective (1959b, p. 84) by seeing autism as onset at birth pseudodefectives. She saw symbiotic psychosis as part of early childhood schizophrenia (1970, p. 166). Additionally, her approach incorporated acute and insidious childhood schizophrenia. She describes how onset “may be insidious and very slow, or abrupt and very rapid” (1947, p. 53). In this regard Bender’s approach did cover a wide range of clinical pictures. However, there were significant limits to its breadth because Bender still demarcated childhood schizophrenia from other disorders. She believed that “differentiation must be made with the neurosis, especially anxiety states and obsessional-compulsive states” (1947, p. 55). Similarly, she demarcated childhood schizophrenia from juvenile delinquents since they had different causal factors: “[t]he nonschizophrenic disturbed children were contrastingly [unlike childhood schizophrenics] reacting to unfavorable homes, interpersonal relationships, and other environmental factors, and their subsequent course could be satisfactorily reversed by controlling these factors” (Bender & Grugett, 1956, p. 142). Childhood schizophrenia can also be difficult to separate from regressive organic disorders but “family history and ultimate course of the disorder can help” (1947, p. 54). However, where psychosis is present then mental deficiency (1959b, p. 85) and organic disorders (1970, p. 166) will be co-morbid with childhood schizophrenia. Psychosis was postulated as needing to be present but different types of psychosis was taken to lead to different clinical pictures.

### Later developments

I have now finished describing the main types of childhood schizophrenia. Further developments would occur in the mid to late 1950s and the 1960s before childhood schizophrenia started to be abandoned in the 1970s. Most of these developments were, however, modifications or alternative understandings of the general framework which was in place by the early 1950s.

Other child psychiatrists held varying views about how all the various diagnoses related to one another. They disagreed over whether these different diagnoses described the same disorder under different names or if different diagnoses represented different stages in the course of childhood schizophrenia. Some child psychiatrists took autism as being early childhood schizophrenia, the autistic child developing into childhood schizophrenia (Alderton, 1966, p. 279; Fish & Shaprio, 1965, p. 42; Havelkova, 1968, p. 851). Others believed autism and childhood schizophrenia tended to merge at age 5 or 6 (Ornitz & Ritvo, 1968, p. 84). Additionally, some child psychiatrists saw symbiotic psychosis as having a relationship to both Kanner’s autism and Bender’s diagnosis. Whereas Mahler saw symbiotic psychosis and autism as different some considered symbiotic psychosis as late onset autism (Eaton & Menolascino, 1966, p. 526) or believed autistic children could develop into symbiotic psychosis (Alderton, 1966, p. 281). Finally, some related symbiotic psychosis to Bender’s approach. Some considered symbiotic psychosis as equivalent to pseudoneurotics or a less severe version of Kanner’s autism and Bender’s pseudodefectives (Fish & Shapiro, 1965, p. 42) or could occur during a transition phase from Kanner’s autism to pseudoneurotics (Havelkova, 1968, p. 852). These child psychiatrists saw autism and symbiotic psychosis as legitimate categories but conceptualised various relationships between them and Bender’s types.

A diagnosis was developed which merged these different approaches. Massachusetts based Beata Rank and Los Angeles based S.A Szurek developed the a-typical child in 1949 (Rank, 1949) and 1956 (Szurek, 1956). They felt all the demarcations employed by other child psychiatrists did not correspond to their clinical experiences, Szurek wrote that.

we are beginning to consider it clinically (that is, prognostically) fruitless, and even unnecessary, to draw any sharp dividing lines between a condition that one could call psychoneurotic and another that one could call psychosis, autism, atypical development or schizophrenia. The concept of a *gradient* of severity of disorder, or that of a psychopathological spectrum is for several reasons becomes for us one which fits our experience most closely. (Szurek, 1956, p. 522)

Consequently, they developed a diagnosis covering all those clinical pictures, including very high and very low functioning individuals. Arguably the concept of the a-typical child was stretched so thin as to be meaningless, but it was a different diagnosis to childhood schizophrenia.

Other child psychiatrists also adopted spectrum type approaches but without, unlike the a-typical child, dispensing with the individual different types. Numerous child psychiatrists conceptualised various disorders as lying on a “continuum” (Robinson, 1961, p. 548; Smolen, 1965, p. 444), others employed the word “spectrum” (Fish et al., 1968, p. 1423), others focused on “gradients” (Esman, 1960, p. 395). Generally, this was conceptualised as a spectrum of severity, whereby Bender’s pseudodefectives and Kanner’s autism were most severe whilst symbiotic psychosis, pseudoneuoretic and pseudopsychopathic were less impaired.

## The abandonment of childhood schizophrenia

The process of abandoning childhood schizophrenia can be traced to four developments in the 1960s and 1970s which I will outline in chronological order. Firstly, there was a reaction to psychoanalytical approaches, especially those which placed emphasis on mother blaming, by parent researchers. Individuals like Clara Park and Bernard Rimland started networks and eventually organizations of parents who objected to psychoanalytical approaches and played a significant role in promoting biological understandings (Vicedo, 2021, p. 112). Relatedly, these parents typically thought that autism was a distinct diagnosis which had no relationship to childhood schizophrenia. Notions of a more narrow autism which was biological in nature started to gain scientific credibility (Vicedo, 2021, p. 112).

Secondly, in 1971 a study was conducted which compared how UK and U.S. psychiatrists diagnosed schizophrenia. It became apparent that UK psychiatrists associated schizophrenia with a narrower clinical picture whereas U.S. psychiatrists associated schizophrenia with a wider clinical picture. This caused a significant controversy and led U.S. psychiatrists to start to move towards a narrower clinical picture (Andreasen, 1989, p. 521). This then made it harder to fit the clinical picture of childhood schizophrenia which I have described into a notion of schizophrenia.

Thirdly, Kanner started the first journal dedicated to research on autism and childhood schizophrenia, *The Journal of Autism and Childhood Schizophrenia*, in 1971. The journal reflected the disunity of the field, with the editorial board consisting of “psychiatrists, psychologists, and psychoanalysts, and the articles that they included in the journal reflected their often sharply divergent approaches to the disorder” (Silverman, 2012, p. 39). However, this all changed in 1978 and 1979. Eric Schopler, who replaced Kanner as editor in 1974, wrote a provocative editorial in 1978. “[S]ince Leo Kanner founded this journal in 1971, many variations of his criteria for infantile autism have been used” (Schopler, 1978, p. 137) but now there are to be “guidelines for reducing confusion [of diagnostic criteria]. It is hoped these will be used for the research published in this journal” (Schopler, 1978, p. 138). This was followed by the new guidelines by Rutter (1978) that set out new diagnostic criteria for autism. These criteria were largely based on Kanner’s 1943 paper (Silverman, 2012, p. 49) except for an emphasis on mental retardation. Of all the various diagnosis employed throughout the past decades only a modified notion of Kanner’s autism would be retained. The journal changed its name to the *Journal of Autism and Developmental Disorders* in 1979. With other journals rejecting psychoanalytical approaches in favour of biological conceptualisations of mental disorder there was soon few places where papers on childhood schizophrenia could be published.

Fourthly, the groundwork for the DSM-III started being developed in the early 1970s by the St Louis group. They wished to increase the reliability of diagnostic categories (Robins & Guze, 1970, p. 108). Childhood schizophrenia was especially vulnerable because there were so many approaches to childhood schizophrenia being employed. They also wished to dispense with (supposedly) speculative causal theories like psychoanalysis (Robins & Guze, 1970, p. 107). Childhood schizophrenia was again especially vulnerable because it was demarcated on the presence of psychosis understood in psychoanalytic terms. Though it had been present in DSM-I and DSM-II, for these reasons older notions of childhood schizophrenia were left out of DSM-III (APA, 1980, p. 86). Childhood schizophrenia was explicitly replaced with DSM-III autism (APA, 1980, p. 86 and p. 375). Meanwhile, the diagnosis of ‘schizophrenia in childhood’ (APA, 1980, p. 375) was added. Unlike notions of childhood schizophrenia, I have described this was heavily associated with hallucinations and thus described a quite different clinical picture.

## Criticism of Eyal et al

Eyal et al. (2010) have heavily criticised the diagnosis of childhood schizophrenia. They claim that “[c]hildhood schizophrenia… [was] stretched so thin as to become meaningless” (Eyal et al., 2010, p. 131), making this claim after discussing Bender’s account of childhood schizophrenia. I will suggest here that Bender’s notion of childhood schizophrenia was more restrictive and specific than Eyal et al. outline.

In evidence of this Eyal et al argue that Bender intended childhood schizophrenia “to cover the whole field of psychiatric interventions from the ‘pseudo-defective’ (mental retardation) to the ‘pseudo-neurotic’ (child guidance) to the pseudo-psychopathic (juvenile delinquency)” (Eyal et al., 2010, p. 131), a “breathtaking sweep of childhood disorders” (Eyal et al., 2010, p. 132). Plausibly, such a diagnosis would be meaningless. For evidence of this claim they quote Bender as saying that childhood schizophrenia was “more common than generally supposed, especially in institutions for mental defectives” (Bender, 1953, pp. 663–664 in Eyal et al., 2010, p. 128). However, this quote does not state the degree, whether high or low, to which Bender thought childhood schizophrenia is more common than previously described. Additionally, Bender specified the presence of psychosis as required, significantly limiting the extent of childhood schizophrenia. To support their claims they also quote Bender as saying that “any severe psychoneurotic disorder in a child before puberty, whether it is obsessive-compulsive, so-called hysterical or simply severe anxiety, is a reactive response to a deeper, inherent threatening disorder, most often schizophrenia” (Bender, 1953, p. 674 in Eyal et al., 2010, p. 128). However, this quote contains the word ‘severe’, which suggests she intended the diagnostic criteria to be restrictive. She is explicit on this point in her 1947 paper (“In each [diagnosed] child it has been possible to demonstrate characteristic disturbances in every patterned functioning field of behavior” (Bender, 1947, p. 40)) and in her 1953 article which Eyal et al focus upon (“The important thing in making a diagnosis of childhood schizophrenia is to realize that one symptom does not make a schizophrenic child; typical symptomatology must pervade in every area of functioning” (1953, p. 673)). Eyal et al. employ neither of these quotes.

I contest Eyal et al’s claims because childhood schizophrenia was more restrictive in who could receive the diagnosis and those who did get diagnosed could have a more specific clinical picture through being considered a particular type of childhood schizophrenia. My history highlights how major child psychiatrists placed significant limits on the extent of childhood schizophrenia through requiring the presence of psychosis. Those child psychiatrists explicitly attempted to demarcate childhood schizophrenia from mental retardation and juvenile delinquency. Also, they conceptualised multiple different types of childhood schizophrenia, some of which were intended to have an associated clinical picture which was different to, even if overlapping with, the other types. Both these points, neither of which are explored by Eyal et al., shows that childhood schizophrenia was less extensive and conveyed more specific clinical information than Eyal et al. believe. Whether childhood schizophrenia is considered too broad or not is a value judgment, but it did not appear to be meaningless or have no clinical use.

## Lessons for thinking about DSM 5 autism

I now consider whether childhood schizophrenia might provide useful lessons for thinking about DSM 5 autism. My stance here is that there is a considerable level of overlap in the symptoms which were associated with childhood schizophrenia and those associated with DSM 5 autism. As such, we can consider whether approaches taken to categorising those symptoms taken between the 1940s to 1970s might have lessons for thinking about how we should categorise the symptoms associated with DSM 5 autism. However, I do not claim that childhood schizophrenia and DSM 5 autism are the same thing. Firstly, the metaphysics of psychiatric diagnoses is highly contestable (Zachar, 2014) whereby we might think there are natural kinds or disease entities out there waiting to be discovered or we might, as I favour (Fellowes, 2022), reject the notion that psychiatric diagnoses should correspond to entities in the world. Secondly, if we did think of in terms of disease entities or natural kinds then it is unclear that DSM 5 autism describes a disease entity or a natural kind given the heterogeneity of causes and symptoms associated with autism. Thirdly, childhood schizophrenia appeared to cover a wider range of symptoms than is associated with DSM 5 autism so if we assume that one of those diagnoses resembles a disease entity or a natural kind then the other resembles it only imperfectly.

### Causes

A number of authors argue that current, symptom-based, approaches to defining autism should be reformulated to be causally based or replaced with alternative diagnoses that are causally based (for example, Cushing, 2013, p. 38; Hassall, 2016, p. 51; Timini et al., 2011, p. 7; Verhoeff, 2013a, p. 9). Thinking about childhood schizophrenia helps us also think about how the diagnosis of autism can be related to causes. In this section I am interested in thinking about different ways of categorising which then results in different ways of relating diagnoses to causes. I am not discussing what are the right or wrong causal descriptions. Childhood schizophrenia was primarily understood psychoanalytically whereas DSM 5 autism is primarily understood cognitively. I have elsewhere analysed and compared both psychoanalytical and cognitive psychological approaches to autism and suggested that both are getting something right, both are getting something wrong and both are ignoring areas that should be covered (Fellowes, 2021a). This is the stance I am also taking in this article.

Whilst there are many causes associated with DSM 5 autism the diagnosis is defined at the level of symptoms. The diagnostician need not detect any particular cause being present in the individual who they diagnose. Also, it is recognised that the diagnosis covers a causally heterogenous population (Petrolini & Vinente, 2022, p. 11; Weiskopf, 2017, p. 178). Whether we consider the genetic, neurological, psychological or social level there appears to be no one cause that is present in all autistic people (Happé & Ronald, 2008, p. 298). Additionally, as I outline, most causes present in autism can be found in people with other diagnoses and in the general population. As such, there appears to only be a loose connection between DSM 5 autism and its associated causes.

In contrast, childhood schizophrenia was causally defined. At least for the sources I have drawn upon, child psychiatrists had to believe psychosis was present for the child to receive a diagnosis of childhood schizophrenia. Additionally, the only relevant factor for receiving a diagnosis of childhood schizophrenia was age and the presence of the cause. This meant that if the cause was taken as present then the child should receive the diagnosis of childhood schizophrenia. Since the cause was considered to manifest in a wide variety of clinical pictures this consequently meant that childhood schizophrenia covered a very broad clinical picture. The clinical picture exhibited by the specific types of childhood schizophrenia was somewhat narrower but the general notion was associated with a very broad clinical picture.

This is worth considering when thinking about causally defining autism. Three cognitive psychological explanations, namely theory of mind deficits (difficulty with seeing other perspectives), weak central coherence (a cognitive style of favouring parts above wholes) and executive dysfunction (difficulty with inhibition and planning), are typically considered the strongest candidates for causally demarcating autism (see Cushing, 2013; Hassall, 2016 for why genetic and neurological factors are even more problematic). As Hill and Frith comment, “[c]ognitive explanations of the core features of autism have provided a vital interface between brain and behaviour” (2003, p. 283). These are intermediate causes whereby biological causes produce these cognitive psychological causes and these cognitive psychological causes then produce symptoms of autism. One way of causally reformulating autism would be to say anyone with one of those causes is autistic. Alternatively, we might split autism up into three subtypes relating to each cognitive psychological cause whereby someone with the particular cause gets the corresponding subtype. However, those causes appear to be present in in non-autistic people. Theory of mind deficits can be present in schizophrenic (Sprong et al.,^2007^, p. 10) and deaf individuals (Paterson, 2016, p. 142). Executive dysfunction can be present in schizophrenia (Orellana & Slachevsky, 2013, p. 5) and ADHD (Happé & Ronald, 2008, p. 298). Weak central coherence can be present in schizophrenia, Williams syndrome, depression, and right hemisphere damage (Happé & Frith, 2006, p. 15). Defining autism in a manner whereby the presence of the cause means presence of autism or the presence of a subtype of autism would radically expand the clinical picture of autism, in an analogous manner to how childhood schizophrenia was causally based but covered a very wide clinical picture. As such, childhood schizophrenia highlights a potential problem with this approach to defining diagnoses based upon causes. We gain a clear way of formulating a diagnostic system, namely if the cause is present then the diagnosis is present, but this risks radically inflating the clinical picture of the diagnosis.

This is far from the only way to define diagnoses based upon causes. Adding additional constraints can prevent the radical expansion of clinical pictures. An approach could be taken whereby to be diagnosed as autistic, or an autistic subtype, one of the three cognitive psychological causes need be present *and* some other specific causes or symptom either need to be present or absent. Perhaps repetitive behaviour needs be present or perhaps hallucinations must be absent. Taking this approach means someone can exhibit one of three main cognitive psychological causes associated with autism and not actually be autistic, thereby not radically expanding the clinical picture of autism. Deciding what additional criteria to employ involves a lot of decision making which will require value judgments. We might value accurately portraying how causes relate to symptoms by basing psychiatric diagnoses around causes regardless of whether this expands the number of symptoms associated with each diagnosis. We might instead value accurately portraying how symptoms cluster together regardless of whether this means diagnoses are causally heterogeneous. Many positions between these are possible by specifying more specific combinations of causes or symptoms as needing to be present or absent. Those interested in causal research will typically value homogeneity of causes. Researchers on factors affecting quality of life might be interested in either approach since internal and external factors can interact with both causes and symptoms. Those interested in medication or therapy might favour either approach since these can target underlying causes or overt symptoms. Those prioritising communicating about patients and administrative decisions over who is eligible for benefits and care might favour basing psychiatric diagnoses around more homogeneous symptoms. Which of these approaches people who receive psychiatric diagnoses favour is an under-researched area but I feel that more homogeneous groupings of symptoms would help diagnosed people find other people who have similar overt characteristics to themselves for purposes of socialising and activism.

This means value decisions are required which then has consequences for critics of the diagnosis of autism. One critic of modern notions of autism says that because autism does not appear to correspond to a natural kind it is “either arbitrary or solely politically/economically motivated” (Cushing, 2013, p. 38). The idea seems to be that making decisions about which psychiatric diagnoses there are, rather than discovering which diagnoses there are through finding natural kinds, means that psychiatric diagnoses are arbitrary or just politically or economically motivated. I disagree with this terminology, having argued that choices in formulating psychiatric diagnoses do not mean arbitrary (Fellowes, 2021b), but if decision making when formulating a diagnosis is a concern then it is important to note that adding constraints when causally formulating diagnoses will also involve decision making. Having formulated a diagnosis around the three cognitive psychological causes associated with autism we then need make decisions if repetitive behaviour is also required for a diagnosis or if the presence of hallucinations means the diagnosis cannot be given. I believe those who criticise the diagnosis of autism on grounds of causal heterogeneity would significantly strengthen their case by outlining what values they would employ and what compromises they would accept when either causally reformulating DSM 5 autism or producing a completely different alternative to DSM 5 autism. Without doing this it is difficult to judge whether and to what degree a diagnostic system that is causally based would be superior to DSM 5 notions of autism.

Childhood schizophrenia, specifically Bender’s approach, also helps us think about how to relate causes to psychiatric diagnoses in other important ways. The causal origins of psychiatric diagnoses are sometimes understood in terms of fixed biological causes. However, individuals typically respond to multiple causes, including environmental ones and ones specific to an individual, both of which may come and go over time. Such specific factors which influence the life story of patients have been largely excluded from DSM research, especially for diagnosis which are considered to have a biological basis (Pietikainen, 2015, p. 323). However, this is a recent direction of research in psychiatry (Hershenberg & Goldfried, 2015, p. 162). In relation to autism, recent research suggests that phenomena like theory of mind should not be understood as simply a dysfunctional internal module. Rather, inter-subjective understanding and perspective taking should be understood as embedded with social communities and wider environmental factors that influence social communication. In this sense, the degree to which an autistic person can be understood as struggling with theory of mind appears to differ depending upon which social community and environment they are in (De Jaegher, 2021, p. 2; Krueger, 2021, p. 21).^4^ Also, their social community and environment can change over time which can then influence what struggles occur or do not occur. Bender’s notion of childhood schizophrenia helps us think through incorporating such factors into psychiatric diagnoses because it was conceptualised as being influenced by specific factors like “individual personality type, the severity and time of onset of the illness, environmental and interpersonal relationships, therapy, the various developmental epochs, severe illnesses, and many lifelong processes” (Bender, 1959a, p. 491). These were considered to potentially alter the defense mechanism. This shows how when causally defining diagnoses it need not be done around static causes that are present throughout the life of the diagnosed individual. We might recognise that each of the three main cognitive psychological causes associated with autism may be present at one stage but not present at another stage of the life of a particular autistic person as the individual ages and their environment changes yet still consider this compatible with causally defining diagnoses.

### Subtyping

A significant difference between historical notions of childhood schizophrenia and DSM 5 autism is the sheer number of different types associated with childhood schizophrenia. Rather than just being diagnosed as childhood schizophrenia someone could instead be diagnosed with a specific type. These types were not simply based upon functioning level. There was not simply ‘high functioning childhood schizophrenia’ and ‘low functioning childhood schizophrenia’, rather, each type of childhood schizophrenia was demarcated on causes and symptoms. In contrast, recent notions of autism subtypes have primarily related to functioning. DSM 5 autism does not have more specific types or subtypes but DSM 5 autism was primarily born out of merging DSM-IV autism and DSM-IV Asperger’s syndrome. Generally, Asperger Syndrome was taken as high functioning whilst autism was taken as low functioning, but they only differed by Asperger’s syndrome not having delayed language whereas autism had delayed language. Meanwhile, there was a notion high functioning autism which was high functioning but did not have delayed language but this notion was not in the DSM-IV. Similarly, there have been recent discussions of adding a notion of profound autism (Singer et al., 2023) which is intended to cover autistic individuals with higher support needs though activists heavily disagree with this notion. This shows how discussions about subtyping autism focus typically on arguing for or against adding subtypes based upon functioning level. In contrast, my history of childhood schizophrenia shows that subtyping does not have to be on level of functioning. Each type of childhood schizophrenia is demarcated in a rich and nuanced manner. Childhood schizophrenia and DSM 5 autism contains many overlapping symptoms so childhood schizophrenia shows how we can take that rough domain of symptoms and formulate multiple subtypes that are not based upon functioning level. What constitutes good subtypes of autism is partly a question of values and partly a question of empirical data. I in no way suggest that the specific types of childhood schizophrenia would be the best or even good subtypes to apply to autism. Rather, my history shows that subtyping in ways that are not based upon functioning levels is fully possible. Also, to my mind, it looks like a more nuanced and promising approach to subtyping than functioning level.

Childhood schizophrenia also suggests the possibility of dynamic subtypes. Bender’s childhood schizophrenia was conceived of as dynamic, whereby diagnosed individuals moved between different types as changes to the psychological cause resulted in changing symptoms. Similar notions are not built into DSM-5 autism even though there is “the possibility of movement within a broad spectrum from low to high functioning through the course of a life” (Walsh et al., 2011, p. 606) and “challenges in social communication and restricted interests and repetitive behaviours may alter in severity levels across time” (Kite et al., 2013, p. 1698). The notion that individuals might change the symptoms they exhibit, and thus the diagnosis they receive, is not present within DSM-5 autism but this is a current innovation in psychiatric research which is known as a heterotypic pattern. This is an area of interest for RDoC [Research Domain Criteria], a major, relatively recent, research program (Cuthbert & Insel, 2013, p. 5; Mittal & Wakschlag, 2017, p. 32). This might be due to changing neurobiology, or psychology, or the individual learning over time, or reflecting over time, or changes to their environment. A system of subtypes would ideally reflect this. Rather than thinking of autism subtypes as static whereby an individual has the same subtype for life we can instead think of sybtypes as dynamic. If the symptoms exhibited by an individual or the causes present in an individual change over time then the subtype of autism changes over time. Bender’s approach show that subtypes can be dynamic to then accommodate changing symptoms or causes over time.

## Conclusion

I have provided a history of U.S. notions of childhood schizophrenia. I have shown the early developments of childhood schizophrenia in the 1930s, the development of different types in the 1930s, 1940s and 1950s and then Bender’s reformulation of childhood schizophrenia in the 1940s and 1950s. I have also shown multiple responses to Bender’s reformulation. I then described the process of abandoning childhood schizophrenia. Most child psychiatrists I have described demarcated childhood schizophrenia from non-childhood schizophrenia and demarcated the different types of childhood schizophrenia from one another. Whilst childhood schizophrenia covered a broad clinical picture I have described had significant restrictions on who received the diagnosis, meaning only a subset of people fitting its broad clinical picture were considered childhood schizophrenic. Also, whilst childhood schizophrenia itself covered a very broad clinical picture, someone diagnosed with childhood schizophrenia could be associated with a more specific clinical picture through being diagnosed with a more specific type of childhood schizophrenia. In this reguard childhood schizophrenia was more restrictive and more specific than is portrayed by Eyal et al. Additionally, Silverman and Raz say notions of autism and childhood schizophrenia were used “interchangeably” (Silverman, 2012, p. 39; Raz, 2014, p. 3). Whilst true of some child psychiatrists, I have outlined how major child psychiatrists demarcated between different types and so did not use them interchangeably. All this gives an important overview into a neglected area of the history of psychiatry and the history of autism.

My history is valuable for thinking about DSM 5 autism. There are significant overlaps in symptoms covered by childhood schizophrenia and DSM 5 autism. As such, we can consider childhood schizophrenia when thinking about formulating alternative diagnoses to cover the symptoms associated with DSM 5 autism. By considering the way in which childhood schizophrenia was causally demarcated I have shown how attempts to causally demarcate autism might increase the clinical picture or require various constraints that might result in various compromises. I believe critics of DSM 5 autism would strengthen their case if they specified what values they endorse and what compromises they would accept. Childhood schizophrenia also shows the possibility of causally demarcating diagnoses upon dynamic causes, the possibility of formulating substantive subtypes that are not simply based on functioning levels and the possibility of an individual changing their subtype over time. When it comes to thinking about alternatives to DSM 5 autism, I believe significant consideration should be given to adding subtypes to DSM 5 autism which are (1) not based upon functioning level but instead based upon a set of symptoms and/or a set of causes, (2) the subtypes are dynamic whereby the subtype exhibited by an individual and any associated causes present in that individual can change over time, (3) the changing subtypes and causes can be influenced by the environment changing and the life history of the individual.

.

## Footnotes

1 Child psychiatrists would sometimes name ‘insidious’ onset as ‘chronic’ onset, including sometimes with in the same paper. Insidious was the more popular word so I shall exclusively employ that.

2 She does not directly say this, rather, those who deteriorate have worse onset and six of the seven acute onset deteriorate whereas only three of the sixteen with insidious onset deteriorated.

3 She does not directly say this but says “inferior levels [of intelligence] are obtained during the phases of regression while they are constant in the chronic [insidious] cases” (Despert, 1938, p. 371). She does not directly link regression with acute onset but since insidious onset are not affected intellectually it seems they do not regress. Therefore it can only be acute onset whom regress.

4 These two references employ enactivist and phenomenological approaches to develop alternative, arguably significantly more sophisticated, alternatives to theory of mind deficits by incorporating how individuals exist in social communities and they interact with external environments. I employ these references because the specific phenomena that theory of mind deficits was intended to cover is also covered by these approaches.
